# Characterization of functional mannose receptor in a continuous hybridoma cell line

**DOI:** 10.1186/1471-2172-13-51

**Published:** 2012-09-12

**Authors:** David J Vigerust, Sherell Vick, Virginia L Shepherd

**Affiliations:** 1 Department of Veterans Affairs Medical Center, VA Medical Center/Research Service, 1310 24th Ave., South, Nashville TN 37212, USA; 2 Department of Pathology, Microbiology and Immunology, Vanderbilt University School of Medicine, Nashville TN 37212, USA

## Abstract

**Background:**

The mannose receptor is the best described member of the type I transmembrane C-type lectins; however much remains unanswered about the biology of the receptor. One difficulty has been the inability to consistently express high levels of a functional full length mannose receptor cDNA in mammalian cells. Another difficulty has been the lack of a human macrophage cell line expressing a fully functional receptor. Commonly used human macrophage cell lines such as U937, THP-1, Mono-Mac and HL60 do not express the mannose receptor. We have developed a macrophage hybridoma cell line (43^MR^ cells) created by fusion of U937 cells with primary human monocyte-derived macrophages, resulting in a non-adherent cell line expressing several properties of primary macrophages. The purpose of this study was to identify and select mannose receptor-expressing cells using fluorescence-activated cell sorting and to characterize the expression and function of the receptor.

**Results:**

In the current study we show that the mannose receptor found on this novel cell has endocytic characteristics consistent with and similar to the mannose receptor found on the surface of monocyte-derived human macrophages and rat bone marrow-derived macrophages. In addition, we demonstrate that these cells engage and internalize pathogen particles such as *S. aureus* and *C. albicans.* We further establish the transfectability of these cells via the introduction of a plasmid expressing influenza A hemagglutinin.

**Conclusions:**

The 43^MR^ cell line represents the first naturally expressed MR-positive cell line derived from a human macrophage background. This cell line provides an important cell model for other researchers for the study of human MR biology and host-pathogen interactions.

## Background

The mannose receptor (MR) is a 175 kDa type I transmembrane protein that was first described by Stahl and coworkers as a cell surface receptor involved in the clearance of extracellular hydrolases [1]. Since that time many more roles have been ascribed to the MR including clearance of pathogens [2], capture of foreign antigens for presentation to MHC-II compartments [3,4], clearance of glycoprotein hormones [5], clearance of extracellular peroxidases [6,7], endocytosis of lysosomal acid phosphatase [8], and regulation of glycoprotein homeostasis [9]. Recent work has suggested that the MR may serve as an entry receptor for several important human pathogens [10-14]. In addition to a cysteine-rich domain and fibronectin type II repeat, the MR structurally contains eight carbohydrate recognition domains (CRD), of which 4, 5 and 7 are reported to be the most critical for binding and internalization of ligands with exposed oligosaccharides terminating in mannose, fucose or N-acetylglucosamine [15].

A characteristic feature of the MR and other members of this family is their rapid internalization from the plasma membrane via a clathrin-mediated mechanism that delivers the receptors to the endocytic pathway [16,17]. Several studies have shown that the MR binds and internalizes ligands via receptor-mediated endocytosis [18,19], and participates in phagocytosis of mannosylated particles and pathogens [20,21]. Mannosylated ligands bind to the MR at the cell surface at neutral pH and are brought into the cell, where they dissociate from the receptor in an acidic endosomal compartment [22,23]. Ligands are then transported to the lysosome for degradation. Degraded particles are either packaged into MHC-II molecules or released into the extracellular media by exocytosis [24]. It has been reported that 10-30% of the receptor at steady state resides on the cell surface and the remaining 70-90% is located in an intracellular pool. The MR has a long half-life (>30 hours), and makes 10 or more rounds of recycling each hour [25].

In addition to endocytic properties, several members of the MR family of molecules participate in phagocytosis, a function vital to the role of the macrophage in the innate immune response. Macrophages are found in virtually all tissues and are among the first cells to encounter an invading microorganism. The recognition capacity of the MR is broad allowing for the capture and uptake of a variety of pathogens including *Paracoccidioides brasiliensis*[26,27]*Candida albicans*[28], *Leishmania donovani*[29], *Mycobacterium tuberculosis*[30,31], *Pneumocystis jirovecii* (*formally Pneumocystis carinii* sp*. hominis*) [32], *Klebsiella pneumoniae*[33], HIV [11,34], Dengue [35], Hepatitis B [36], and influenza A [10,37]. Mannose is not often found as a terminal residue on the cell surface of mammalian-derived proteins, but is commonly found on the surface of a variety of pathogens, thus allowing the MR to distinguish self from non-self through carbohydrate recognition.

Expression and function of the MR is tightly linked to the activation state of the macrophage and a variety of cytokines and chemical messengers are involved in the regulation of the MR. For example, interleukin (IL)-4, IL-10, IL-13 and prostaglandin E_2_ (PGE_2_) are known to up-regulate the expression of the MR [38-41], as does exposure to dexamethasone and vitamin D_3_[42-44]. Proinflammatory cytokines that activate macrophages at sites of inflammation such as interferon (IFN)-γ down-regulate MR expression [45]. Exposure to pathogens such as *Leishmania*, *C. albicans,* bacillus Calmette-Guerin, HIV-1, and influenza likewise down-regulate receptor expression [46,47]. This complex system of regulation is critical to the role that the MR plays in the resolution of inflammation, allowing for efficient removal of harmful extracellular enzymes such as myeloperoxidase, eosinophil peroxidases, tissue plasminogen activator, and lysosomal hydrolases [6]. Further support for an *in vivo* role for the regulation of extracellular glycoproteins has come from studies with MR null mice that showed that the lack of MR results in decreased clearance of hydrolases and procollagens [9].

To date there are very few macrophage cell lines that express a functional MR. Commonly used human macrophage cell lines such as U937, THP-1, Mono-Mac and HL60 do not express the MR. Those cell lines that have been described as MR-positive are of murine or rat derivation [45,48,49]. In particular the MR-positive murine J774E and rat NR8383 cell lines have been extensively used in studies of MR function but until the current study there has been no available continuous human macrophage cell line that expresses the MR.

The MR has been implicated as a potential entry receptor for a variety of pathogens, and a target for regulation by human pathogen-associated proteins suggesting relevance for this receptor in the context of human disease [10,11,50]. Additionally, the glycosylation profile of many human pathogens makes uptake via the MR likely [37,51-53]. In the current study we have characterized and cultivated a human hybridoma cell line that expresses MR. The utility of a human macrophage cell line and expression system for the MR will allow further study of MR biology and characterization of host-pathogen interactions between macrophages and causative agents of infectious disease. These tools will allow for further study of the biology and trafficking of the MR within the cell.

## Methods

### Cells

Macrophages used in this study included rat bone marrow-derived macrophages (RBMM), U937, and the continuous human macrophage hybridoma cell line 43 (obtained from Dr. K. Sperber, Mt. Sinai Medical Center), generated by fusing the hypoxanthine-guanosine phosphoribosyltranferase-deficient promonocytic cell line, U937, with human monocyte-derived macrophages (MDM). Fusion was documented by the acquisition of donor class I molecules [54]. U937, 43 parental cells, and 43^MR^ cells were maintained in RPMI 1640 with 10% FBS and antibiotics. RBMM were prepared from bone marrow obtained from Sprague–Dawley rats as previously described [55,56] and maintained in RPMI with 10% FBS and antibiotics.

### Plasmids and antibodies

The polyclonal antibody used in immunoblot analysis for the detection of MR was prepared in our laboratory [49]. Monoclonal antibody against the human MR was obtained from BD Biosciences as an unlabeled antibody and also directly conjugated to Alexa 488 (San Jose CA). Mouse anti-hemagglutinin (HA) directly conjugated to Alexa 488 was obtained from Invitrogen (Carlsbad CA). Anti-EEA1 monoclonal antibody was obtained from BD Biosciences (San Jose CA). Antibodies conjugated to Alexa dyes (488, 555, and 568) for use in cytometry studies were obtained from Invitrogen (Carlsbad CA). Phycoerythrin (PE)-conjugated antibodies for use in fluorescent-activated cell sorting (FACS) were purchased from BD Biosciences (San Jose CA). Influenza PR8 HA plasmid was obtained from Adolfo Garcia-Sastre (Mt. Sinai Medical Center) and used for transfection assay to determine the capacity and efficiency of transfection.

### Transfections

To determine the efficiency of transfection, 43^MR^ cells were transfected with Fugene (Roche) at a ratio of 6:1 according to manufacturer directions. Cells at 1 x 10^6^ cells per well were plated into 6-well tissue culture dishes and transfected with 5 μg of PR8 HA plasmid. Cells were incubated at 37°C for 24 hours, and then harvested for flow cytometry as previously described.

### Flow cytometry and confocal microscopy

Flow cytometry was performed using a FACSCaliber (BD Biosciences, San Jose CA) bench top analyzer in the VA and Vanderbilt cytometry core facilities. Cells were stained with PE-conjugated antibodies against MR as follows: cells were collected by centrifugation, followed by suspension in staining buffer (1% BSA, 0.1% sodium azide, 0.5% normal goat serum in PBS). 10% normal goat serum was included in the stain buffer to saturate Fc receptors and minimize non-specific fluorescence. PE-conjugated antibody was diluted in staining buffer and added to the cells. Additionally, an isotype matched control was also performed to account for non-specific fluorescence, and an unstained sample was included to account for autofluorescence. The cells were incubated for 20 minutes in the dark at 4°C. The cells were washed twice with staining buffer followed by fixation in 500μl of 2% buffered paraformalin. The modulation of cell surface receptor density was represented as the percent change in mean fluorescence intensity as compared to the control. The number of events acquired for each sample was 3 x 10^4^ and cells were analyzed on a Becton Dickinson FACScan flow cytometer using CellQuest software or WinMDI FACS analysis software from J. Trotter (Scripps Research Institute, La Jolla CA). Characterization of the cell surface expression profile of the 43^MR^ cells was performed by flow cytometry using antibody against typical macrophage surface expressed molecules (HLA ABC, CCR5, CD32, CD64, CD4, CD14, transferrin, DC-DSIGN, CXCR4, CD80, CD86, CD11) obtained from BD Biosciences (San Jose, CA). Confocal microscopy was performed using a Zeiss LSM-510 laser scanning inverted microscope. Cells were fixed in 2% buffered paraformalin followed by permeabilization with ice cold methanol. Following fixation and permeabilization, cells were stained in two ways. First, a two-step process using mouse monoclonal anti-MR antibody (BD Bioscience, San Jose CA) followed by either goat anti-mouse Alexa-488 or Alexa-568 (Invitrogen, Carlsbad CA) secondary. Second, a custom mouse monoclonal antibody against MR directly conjugated to Alexa 488 was also used. This second method eliminates the potential problem of non-specific secondary binding. Nuclei were stained for visualization with TO-PRO-3 (Invitrogen, Carlsbad CA). Cells were mounted in Aqua-polymount (Polysciences, Warrington PA) between coverslips. Images were collected at 520 nm using a 63x oil-immersion objective. Image processing was performed using Adobe Photoshop CS imaging software.

### Phagocytosis assay

Phagocytosis was assayed via uptake of pHrodo BioParticles (Invitrogen, Eugene, OR). pHrodo *S. aureus* BioParticles conjugates are novel, no-wash fluorogenic particles used for quantitative measurement of phagocytosis. Since the fluorescent intensity of the bioparticle is tightly linked to the pH of the environment, particles in the extracellular environment typically display little to no fluorescence. The resulting fluorescence can therefore be attributed to internalized particles. Cells were cultured in complete media at a concentration of 5 x 10^5^ in 6-well tissue culture dishes. After 24 hr, BioParticles prepared according to the manufacturer’s instructions were added to cells, and the plates incubated at 37°C for 30 minutes to allow for uptake. Cells were washed to remove unattached BioParticles, followed by fixation in 2% paraformalin and staining with anti-MR Alexa-488 conjugated antibody, and then visualized by confocal microscopy. In addition to examining the phagocytosis of labeled *S. aureus*, uptake of GFP-*Candida* was also employed. GFP-*Candida* was provided by L. Hoyer (U of Illinois) and incubated with cells in a 5:1 ratio at 37°C for 30 minutes. Samples were examined by confocal microscopy using anti-MR Alexa 568- or Alexa 555-conjugated antibody as described above.

### Imunoprecipitation and immunoblot analysis

Cells (5 x 10^5^) were lysed in lysis buffer containing 0.5% NP-40 or 0.1% Triton. Total cellular protein was determined either by bicinchoninic acid (BCA) protein assay (Pierce, Rockford IL) or by NanoDrop spectrophotometer (Thermoscientific) and 10-25 μg of total protein was resolved by electrophoresis on a 7.5 or 10% SDS-PAGE gel. Proteins were transferred to nitrocellulose followed by blocking in Tris-buffered saline (TBS) containing 0.1% Tween (TBST) and 5% powdered milk. Proteins were visualized on radiographic film using primary antibodies to the desired protein, horseradish peroxidase (HRP)-conjugated secondary antibodies, and chemiluminescence reagent. Immunoblot analysis was also performed on the Licor Odyssey platform using a goat anti-MR antibody followed by donkey anti-goat Ir-680 dye and a mouse anti-actin antibody followed by a donkey anti-mouse Ir-800 dye (Lincoln NE). For immunoprecipitation, cells were seeded into 6-well dishes at 5 x 10^5^ cells per well and treated accordingly. Cells were lysed in 0.5% NP-40 lysis buffer and lysate was precleared with protein A-Sepharose overnight at 4°C followed by immunoprecipitation using rabbit polyclonal antibody against MR. The immunoprecipitated receptor was detected by immunoblot analysis after electrophoretic separation on 7.5% SDS-PAGE gel. Bands were visualized by chemiluminescence and exposure of Kodak Bio-MAX film. The density of the bands was quantified using the UN-SCAN IT densitometry software from Silk Scientific (Orem UT).

### Receptor internalization

Receptor internalization was assessed using FITC-Man-BSA, FITC-BSA or fluorescent-labeled antibodies in the following manner: triplicate samples of 5 x 10^5^ cells were pelleted in 12 x 75 mm Falcon tubes (Becton Dickinson Labware, Franklin Lakes NJ), washed twice in cold PBS to remove all media, and repelleted. The cells were placed on ice and a 1:100 dilution of anti-human MR conjugated to PE in cold staining buffer was used to resuspend the cells. Cells were incubated on ice for 15 min. Cells were washed twice in cold PBS and samples were taken to represent the total binding. Cells were then warmed to 37°C and triplicate samples were taken at 0, 10, 20, 30 minutes and placed on ice. Cells were pelleted and fixed in 500 μl cold 2% paraformalin containing 0.1% trypan blue to quench extracellular fluorescence. Cells were then analyzed by flow cytometry for total fluorescence. Fluorescence representing surface MR was plotted versus time to determine receptor internalization.

### Uptake of horseradish peroxidase (HRP)

To measure ligand internalization, 10 μg of HRP was incubated with 43^MR^ cells (5 x 10^5^) in 400 μl Hanks’ balanced salt solution (HBSS) containing 1% BSA (binding buffer) as described previously [57,58]. To control for non-specific uptake, yeast mannan (Sigma, St. Louis MO) was added at 1 mg/ml to companion wells as a competitor to HRP. Cells were incubated for 60 minutes at 37°C. Following incubations, the cells were washed twice in HBSS and solubilized in 250 μl of 0.1% Triton X-100. Cell-associated activity was determined by quantifying the oxidation of phenol red in the presence of hydrogen peroxide. HRP activity was then normalized to total cellular protein as measured by the BCA protein assay.

### Ligand binding

#### Binding of fluorescein isothyocyanate mannosylated-BSA (FITC-Man-BSA)

FITC-Man-BSA and FITC-BSA (20 μg) (E-Y laboratories, San Mateo CA) was incubated with 43^MR^ cells (1 x 10^6^) in 100 μl of staining buffer in the presence or absence of 1 mg/ml mannan. Incubations were done in triplicate and were incubated on ice for 30 minutes. Following incubation, cells were washed twice in cold staining buffer, fixed in 2% paraformalin and analyzed by flow cytometry.

#### Binding of ^125^ I-mannosylated-BSA binding (^125^I-Man-BSA)

Man-BSA was iodinated to a specific activity of 2 x 10^8^ cpm/μg as previously described [19]. Cells (5 x 10^5^) were incubated with ^125^I-Man-BSA in 100 μl of binding buffer in the presence or absence of yeast mannan for 60 minutes on ice. Cell-associated counts were separated from unbound ligand via centrifugation through mineral oil, and cell-associated ligand was quantified by gamma counting.

### Immunoprecipitation of ^35^S-labeled receptor

Cells were seeded into 6-well dishes at 5 x 10^5^ cells per well and labeled by incubation with 100 μCi of ^35^S-methionine (Amersham, Piscataway NJ) in methionine-free DMEM overnight. Labeled receptor was immunoprecipitated using polyclonal anti-MR antibody as previously described [59]. The immunoprecipitated receptor was analyzed on 7.5% SDS-PAGE gels. Bands were visualized by exposure of Kodak Bio-MAX film. The density of the bands was quantified using a scanner and UN-SCAN IT densitometry software.

### Reverse transcriptase-polymerase chain reaction (RT-PCR) analysis of MR cDNA

RT-PCR of the 650 bp fragment was performed with a sense primer derived from rat and murine cDNA. The primer sequences were as follows: sense oligonucleotide (S3): 5’-GCTCTAGAATGGAACACACACTCTGGGCCATG-3’, antisense oligonucleotide (650AS): 5’-TACCACTTGTTTTCAAACTTGAA-3’. RT-PCR of the 300 bp cytoplasmic tail fragment (TMC) was performed with sense and antisense primers derived from the rat MR cDNA: sense oligonucleotide (TMCR): 5’-GAGACAGTCAGATTAGGATGGCTAA-3’ antisense oligonucleotide (TMCL): 5’-AAAGCTGACCAAAGGAAGATG-3’. PCR reaction conditions were as follows: total RNA was obtained from cell populations using Trizol and reverse transcribed using the First-Strand cDNA synthesis kit (Pharmacia, Piscataway, NJ). The PCR reaction mixture contained 1 μl of MR primer (100 ng/μl); 1 μl of a 20 mM dNTP solution; 5 μl of 100 mM Tris buffer with 15 mM MgCl_2_ and 500 mM KCl (pH. 8.3); 1 μl of template cDNA; 1 μl of Taq DNA polymerase (Promega); and 40 μl of diethlypyrocarbonate (DEPC) treated water. Thirty cycles of amplification were performed in a Hybaid OMN-E thermocycler (MR 650: 5 minutes at 92°C, 30 seconds at 92˚, 30 seconds at 48°C, and 1 minute at 68°C; MR TMC: 5 minutes at 95°C, 1 minute at 95°C, 1 minute at 55°C, and 1 minute at 72°C). Aliquots of the PCR reactions were then analyzed on agarose gels, and bands were visualized by ethidium bromide staining.

### Statistical analysis

Statistics were performed using Prism 5 (GraphPad Software). For experiments involving two comparisons, a paired Student’s two-tailed *t*-test was used. For experiments involving in excess of two comparisons, a one way ANOVA with post-test was performed. Significance was determined by p values less than 0.05. All experiments presented are representative of at least 3 determinations with error bars that represent the standard deviation unless otherwise noted in the figure legends.

## Results

### Selection of MR-positive 43 cells

**Figure 1:**
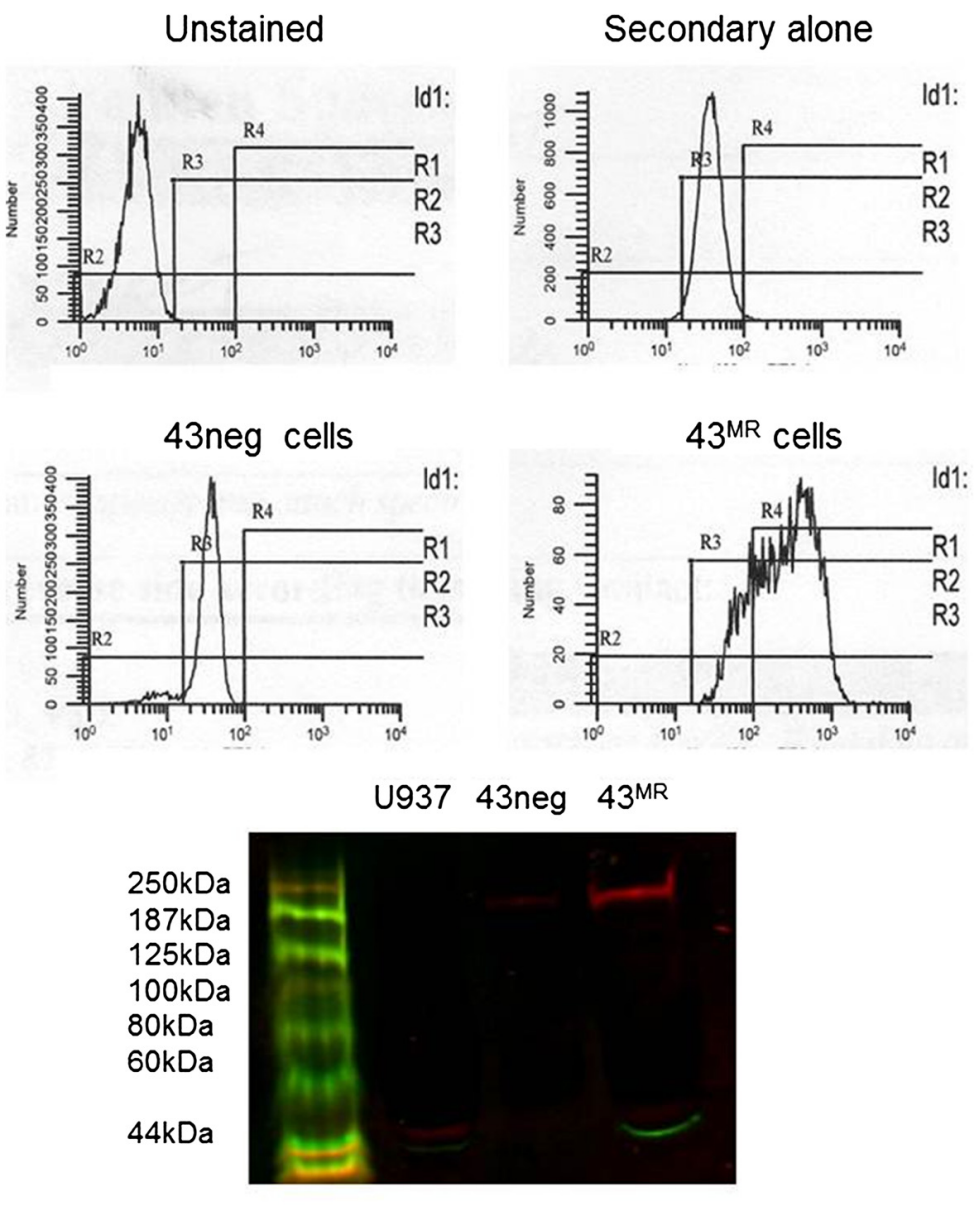
**Flow cytometric analysis of parental and MR-positive Cells. **43, U937 and 43MR cells were harvested and prepared for Western blot and flow cytometric (FACS) analysis. For FACS analysis, cells (1 x 10^6^) were harvested and fixed in 2% paraformalin. As negative controls, both 43 and 43MR cells were stained with Alexa 488 alone (upper right histogram) or unstained (upper left histogram). For detection of MR, 43 cells or 43^MR^ cells were stained with mouse monoclonal anti-MR followed by Alexa 488 conjugated goat anti-mouse secondary as previously described. The number of events acquired for each sample was 3 x 10^4^. Data shown are the relative change in mean fluorescence intensity as compared to the control, and data are representative of at least two independent experiments. For Western blot analysis, 1 x 10^6^ cells were lysed in 0.1% Triton lysis buffer and 25 μg of total protein was separated by SDS gel electrophoresis. Proteins were transferred to nitrocellulose and the blot probed with goat anti-MR and mouse anti-actin antibodies followed by Licor donkey anti-goat Ir-680 and donkey anti-mouse Ir-800 conjugated secondary antibodies.

MR-positive 43 cells (43^MR^) were selected as described below. Upon initial analysis the parental 43 cells were negative for MR expression. For induction of MR expression, cells were cultured in an equal mix of fresh and spent/conditioned media (RPMI 1640 plus 10% FBS) over a 12 month period. During this period, cells were harvested and repeatedly sorted for MR expression and MR negative cells were discarded. The resulting cell line – greater than 95% MR positive - was termed 43^MR^. Figure 1 shows a typical FACS analysis and western blot of MR expression during the selection process. Mean fluorescent intensity of 43^MR^ increased by 7-fold over 43 cells. Protein lysate was analyzed for MR expression (red) and also was analyzed for the expression of a loading control. The β-actin band (green) is seen clearly in the 43^MR^ and U937 lanes but not as clear in the 43neg cells. We have performed nearly a dozen immunoblot assays using both β-actin and GAPDH as loading controls. Although these are commonly used for loading controls, there are data to suggest that these proteins are not ideal in many situations [60] and a total protein approach is more appropriate. We have run a constant amount of total protein in our immunoblot. Data presented here shows β-actin bands in all three lanes but not at the same intensity. This was also the case with GAPDH. Although the intensity is not identical we feel that the wells were equally loaded and accurately reflect the content of the protein lysate.

### Cell surface expression characteristics of the 43^MR^ macrophage hybridoma cell line

Macrophages express a variety of cell surface molecules that participate in the identification and removal of intracellular and extracellular molecules. However, surface expression in cell lines varies, and can be quite different from primary macrophages. To determine the cell surface profile of 43^MR^ cells, cells were stained with a variety of common macrophage cell markers. Table 1 shows the cell surface expression profile of the parent 43, 43^MR^, U937, and primary MDM. Similar to primary macrophages, 43^MR^ cells expressed HLA ABC, CCR5, CD32 and CD64, and was the only cell line expressing the MR. It should be noted that 43^MR^ cells do not express CD80, CD86, DCSIGN, or CXCR4, all of which are expressed at low levels by MDM but are not present on U937 cells, suggesting that during the fusion process these specific cell surface proteins were not induced. Figure 1 demonstrates the surface profile by FACS analysis of 43 negative parental cells (43neg), U937 and the 43^MR^ cells. Figure 1 further shows by Western blot that the U937 and 43neg cells are negative for MR expression while the 43^MR^ cells show a strong band at approximately 175 kDa.

**Table 1:** Cell surface expression profile of macrophages and macrophage cell lines

**Marker**	**MDM**	**U937**	**43**	**43**^**MR**^
**HLA ABC**	**+++**	**+++**	**-**	**++**
HLA DR DQ	+	+++	++	+/−
CD4	+	+++	++	+/−
CD11b	+	+/−	n.d	+/−
CD11c	+	-	n.d	+/−
CD14	+++	+++	n.d	+
CD32 (FcRγIII)	++	+++	n.d	+
CD36	n.d	+	n.d	+/−
CD64 (FcRγI)	+	-	n.d	+/−
CD71 (TfR)	n.d	+++	n.d	+++
CD80	+	-	++	-
CD86	+	-	++	-
**CD206 (MR)**	**+++**	**-**	**-**	**+++**
DC SIGN	+	-	n.d	-
CCR5	+	n.d	n.d	+/−
CXCR4	+	+	n.d	-
Dectin	+	n.d.	n.d.	+

Cells (1 x 10^6^) were harvested and stained with a variety of antibodies against cell surface markers to determine the expression character of the 43^MR^ cells. Cells harvested for unstained and negative control samples were incubated with an isotype matched control to obtain baseline fluorescence. Cells were analyzed via FACS and scored as the percentage of positive cells above isotype control as follows: - (0-5%), +/− (6-25%), + (26-50%), ++ (51-75%), +++ (76-100%). n.d = not determined/unavailable. Data for macrophages can be found reviewed in 43 cells in Sperber et al. [54] represents published cell surface information and data for 43, 43^MR^, and U937 cells represent at least three independent experiments.

### Expression of the MR protein by 43^MR^ Cells

**Figure 2:**
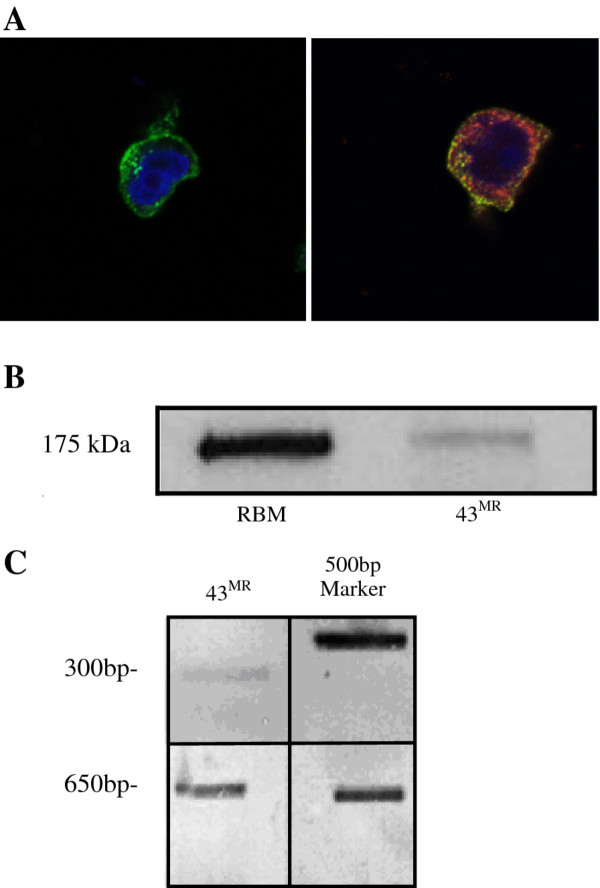
**Imaging, immunoblot and RT-PCR analysis of MR protein expression from macrophage cells. **In panel **A**, 43^MR^ cells were processed for confocal imaging as described in Methods. Cells were fixed in 2% buffered paraformalin for 15 minutes and permeabilized in ice cold methanol for 10 minutes. Cells were then washed and stained with mouse anti-MR directly conjugated to Alexa 488 and rabbit anti-EEA1 antibody followed by goat anti-rabbit Alexa 568 conjugated secondary. Nucleus was stained with TO-PRO 3 for visual reference. (**B**) RBMM and 43^MR^ cells (1 x 10^6^) were harvested and lysed in NP-40 lysis buffer for immunoblot analysis. Proteins (10 μg) were resolved on a 7.5% SDS-PAGE gel. Immunoblot analysis was performed with a polyclonal antibody against the MR and HRP-conjugated secondary antibody. Detection of protein was performed by enhanced chemiluminescent assay and exposure of Kodak Bio-Max scientific film. (**C**) Total mRNA was prepared from 43^MR^ cells (1 x 10^6^) using Trizol reagent, and reversed transcribed using the First-Strand cDNA kit. The cDNA preparations were then subjected to PCR using the 5’ and 3’ primers described for the 650 bp and 300 bp fragments. The data are representative of three independent experiments.

Expression of the MR protein was determined by confocal microscopy and immunoblot analysis to corroborate the flow cytometry data showing cell surface expression of the MR. Whole cell lysate (10 μg) from 43^MR^ cells and control RBMM was resolved by SDS-PAGE electrophoresis. Confocal microscopy demonstrated that the MR protein resides on the surface as well as in discrete intracellular compartments where it localizes with EEA1 consistent with the description of MR in primary macrophages (Figure 2A). Co-localization with EEA1 indicates that the MR is trafficking thru endosomal compartments. For immunoblot analysis, whole cell lysate (10 μg) from 43^MR^ and control RBMM was resolved by SDS-PAGE electrophoresis. Using a polyclonal antibody generated in our laboratory from human placenta, results showed a 175 kDa band (Figure 2B), with expression of the MR in 43^MR^ cells approximately 10-fold less than control RBMM by densitometric scanning. This level of MR expression in 43^MR^ cells compared to RBMM (1:10) is in agreement with estimates of levels of the MR in primary human macrophages [61]. The affinity of this antibody for human, mouse and rat MR has been tested in our laboratory and we find that the antibody equally recognizes MR from these different species.

### Reverse transcriptase polymerase chain reaction (RT-PCR)

Expression of MR mRNA was assessed by RT-PCR using oligonucleotides derived from the rat MR cDNA sequence [49]. The primers used generated an internal 650 bp fragment within the extracellular region of the MR and a 300 bp fragment from the C-terminal end of the cytoplasmic tail of the MR. Figure 2C demonstrates that both a 650 bp and 300 bp fragment was seen in the 43^MR^ cells, consistent with previously published data from RBMM [49], and was not detected in parental 43 cells (data not shown).

### Mannose receptor turnover in 43^MR^ cells

**Figure 3:**
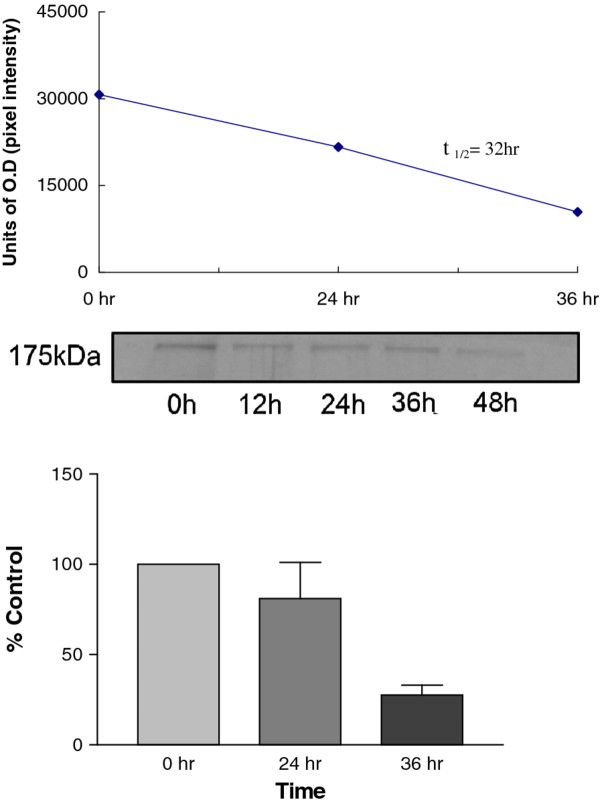
**Mannose receptor turnover in macrophage hybridoma cells. **43^MR^ cells (1.5 x 10^7^) were labeled with 1 μCi of ^35^S -methionine overnight in methionine-free media. Following overnight incubation the cells were washed twice with PBS. Cells were then resuspended in complete media and plated into 6 well dishes. Cells were harvested at designated times and lysed in IP buffer in preparation for immunoprecipitation. Immunoprecipitates were resolved on a 7.5% gel and transferred to nitrocellulose. Relative pixel intensity of bands was determined by densitometry. Data represents the percent change in relative pixel intensity from the control at time 0. Results are representative of three independent experiments.

In this study we have shown by FACS that the MR is expressed on the surface of 43^MR^ cells and that the receptor is the same molecular weight as the MR from RBMM by immunoblot analysis. To further characterize the MR in 43^MR^ cells, the half-life in comparison to MR turnover in RBMM was examined. Cells were metabolically labeled with ^35^S-methionine followed by immunoprecipitation and analysis by immunoblot and densitometry. The results indicated that the MR in 43^MR^ cells has an approximate half-life of 32 hours (Figure 3) which is consistent with published data on the RBMM MR [49].

### Binding and uptake of ligands mediated by the MR in 43^MR^ cells

**Figure 4:**
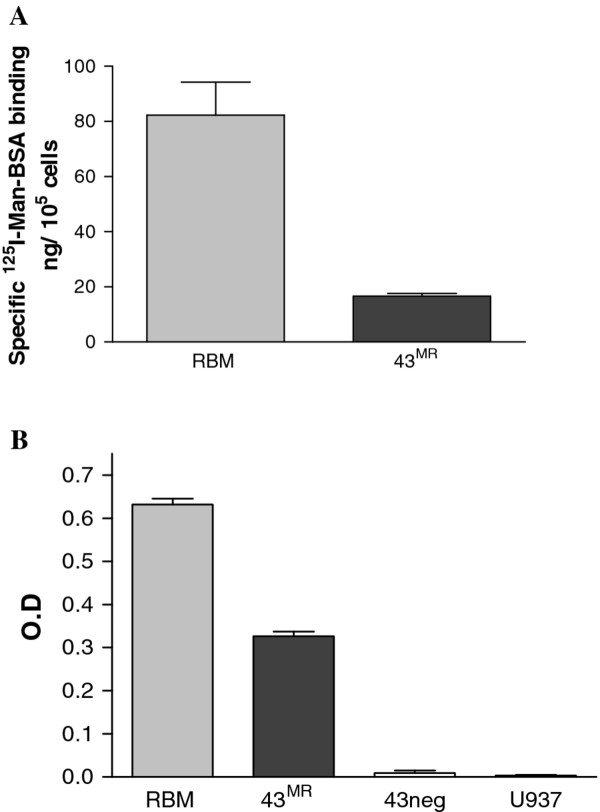
**Comparison of specific surface binding and HRP uptake in RBMM and 43**^**MR **^**cells. **(**A**) Cells (5 x 10^5^) were incubated on ice for 60 minutes with approximately 0.4 μg of ^125^I-Man-BSA in 100 μl of binding buffer in the presence or absence of mannan (1 mg/ml). Cells with bound ligand were separated from the unbound ligand by centrifugation thorough oil. Cell-associated radioactivity was quantified by gamma counting. Results are representative of three independent experiments. (**B**) RBMM, U937 43neg and 43^MR^ cells were incubated with 8 μg of HRP in 400 μl of Hanks’ balanced salt solution (HBSS) containing 1% BSA in the presence or absence of mannan (1 mg/ml). Cells were incubated at 37°C for 60 minutes, then washed twice in HBSS and solubilized in 0.1% Triton X-100. Cell associated HRP activity was determined by quantitation of phenol red oxidation in the presence of hydrogen peroxide at 610 nm.

**Figure 5:**
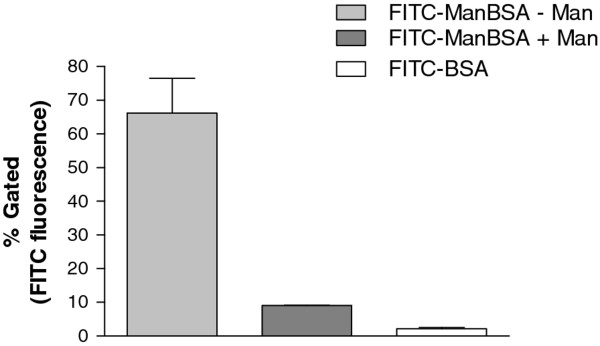
**Flow cytometric analysis of 43**^**MR **^**uptake of FITC-Man-BSA.** 43^MR^ cells (1 x 10^6^) were seeded in 6-well tissue culture dishes and incubated with FITC-Man-BSA in the presence or absence of excess mannan (1 mg/ml) at 37°C. Cells were also incubated with FITC-BSA as a negative control. Following incubation, the cells were harvested and fixed in 2% paraformalin for FACS. Cells were also harvested for unstained control samples. The number of events acquired for each sample was 3 x 10^4^. Data shown are the relative change in mean fluorescence intensity as compared to the control. Data are the mean +/− S.D. of at least two independent experiments.

Previous work has shown that the cell surface macrophage MR mediates binding and uptake of a variety of mannose containing proteins [62-65]. To characterize these properties of the MR in 43^MR^ cells, binding of ^125^I-Man-BSA (Figure 4A), uptake of HRP (Figure 4B), and uptake of FITC-Man-BSA and FITC-BSA (Figure 5) was examined. An analysis of the surface binding was performed using radiolabelled Man-BSA to determine the level of surface expression. RBMM were used as a control for comparison. In each case, 5 x 10^5^ cells were incubated with ^125^I-Man-BSA for 60 min at 4°C in the presence and absence of excess mannan to determine non-specific binding. As shown in Figure 4A, RBMM bound approximately 60 ng versus 10 ng by 43^MR^ cells, suggesting that the 43^MR^ cells express fewer receptors on their surface. These results agree with previous findings from our group that human macrophages have lower surface levels of MR than murine or rat macrophages [61] .

The MR participates in the internalization of a variety of glycosylated proteins. HRP is a known ligand for the MR and has been used extensively to examine the endocytic capacity of the MR. To determine the endocytic activity of the MR in 43^MR^ cells, uptake of HRP was measured in and compared to the uptake in RBMM, U937 and the 43neg parental cell line in the presence and absence of mannan. As shown in Figure 4B, 43^MR^ cells efficiently ingested HRP, with uptake of ~50% of RBMM activity. The MR negative cells 43neg and U937 did not internalize a significant amount of HRP. These results indicate that the MR in 43^MR^ cells can mediate uptake of traditional ligands.

Several investigators have adopted the use of flow cytometry to determine the endocytic capacity of membrane-associated receptors [66]. Results can be obtained by using either tagged ligands or tagged antibodies raised against the receptor. We used a FITC-labeled ligand specific (Man-BSA) for the MR to determine the level of uptake exhibited by 43^MR^ cells in the presence of absence of same specific competitor (Man). The fluorometric approach has greater sensitivity than the HRP approach and is further improved by having a ligand with higher avidity (Man-BSA) than HRP. Our results indicate that approximately 60% of cells specifically internalized FITC-Man-BSA (Figure 5). Control experiments using FITC conjugated to a non-mannosylated BSA was also performed to demonstrate that the FITC-BSA ligand itself is not internalized by these cells. Therefore, this uptake was specific for the MR as evidenced by the reduction in internalization in the presence of excess mannan (Figure 5).

### Phagocytic activity of 43^MR^

**Figure 6:**
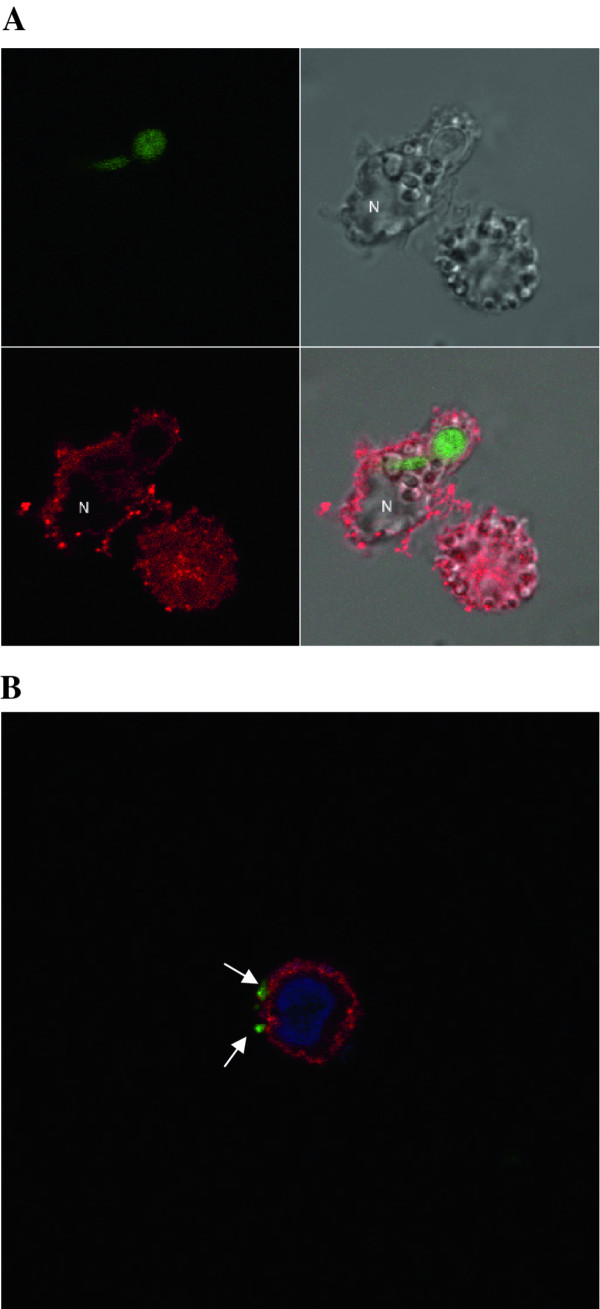
**Phagocytic uptake and binding of pHrodo-labeled**-***S. aureus *and GFP-*Candida albicans.*** 43^MR^ cells (1 x 10^6^) were seeded in 6-well tissue culture dishes and incubated with either GFP-labeled *Candida albicans* or pHrodo *Staphylococcus aureus* for 30 minutes at 37°. Following incubation, the cells were washed twice in PBS and fixed in 2% paraformaldehyde. Following fixation, the cells were stained for MR using the previously described monoclonal antibody. Images were collected by confocal microscopy. N, nucleus. Arrows indicate internalized particles.

Although there have been claims that the MR is not a phagocytic receptor [67], the majority of reports suggest that the MR is intimately involved in the binding and internalization of particulate ligands. To examine the phagocytosis of MR particulate ligands by 43^MR^ cells, cells were incubated with either pHrodo-conjugated *S. aureus* or GFP-labeled-Candida. pHrodo *S. aureus* particles were found to bind to the cell surface and were internalized (Figure 6B). The cells incubated with pHrodo labeled bacteria were found to contain between 3–20 bacterial particles per cell as determined by Z-sectioning and counting of engaged and internalized particles. The fluorescent intensity of internalized particles increases as the pH of the intracellular compartment increases. Figure 6A demonstrates that the much larger yeast particle is internalized by 43^MR^ cells; yeast particles are clearly visualized within the cytoplasm of the cell.

### Transient transfection

**Figure 7:**
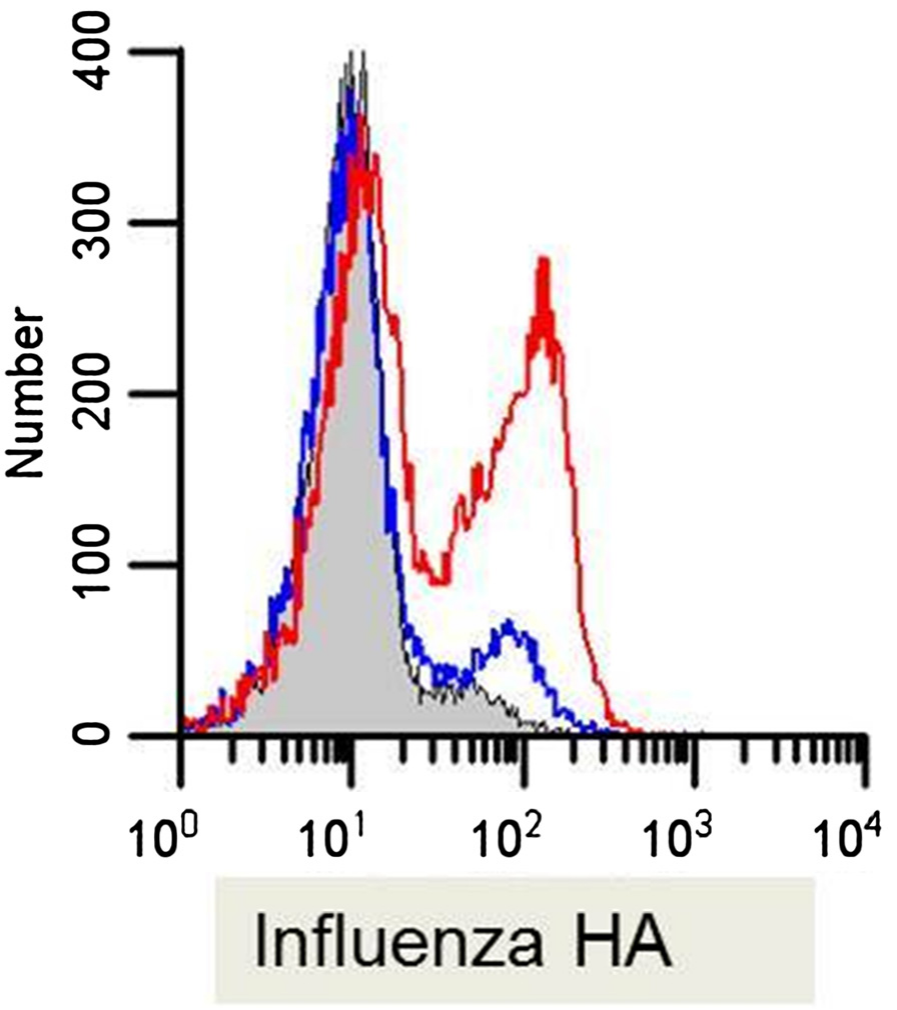
**Transient transfection of influenza A hemagglutinin in 43**^**MR **^**cells. **43^MR^ cells (1 x 10^6^) were seeded in 6-well culture dishes and transfected with 5 μg of Influenza A PR8 HA expressing plasmid. Cells were then incubated for 24 hours at 37. Following incubation, the cells were harvested and stained for flow cytometry with Alexa 488 conjugated anti-HA (red line). Cells were also harvested for unstained (shaded gray) and transfection media alone control samples (blue line). The number of events acquired for each sample was 3 x 10^4^. Data shown are the mean fluorescence intensity as compared to the unstained and transfection media alone control.

Primary macrophages are notoriously difficult to transfect. One of the benefits to a human cell line that expresses the MR is the use of transfection to express proteins to study their interactions with the MR. In the current and previous studies we have examined the effect of several viral proteins in these cells to demonstrate active regulation of the MR by viral proteins [12]. Figure 7 demonstrates the expression of influenza A PR8 HA protein on the cell surface of the cell. At 24 hrs post transfection, 40% of the cells are expressing HA on the cell surface. Transfected cells were stained and examined alongside untransfected (transfection media alone) and unstained cells. A small amount of autofluorescence is seen in these cells and is evidenced by the small peak to the right of the unstained control. Similar transfections have been conducted with proteins from rotavirus, HIV and influenza with efficiencies in the 40-70% range (data not shown).

## Discussion

Macrophages are critical members of the innate immune system, and play a pivotal role in the clearance of pathogens, foreign particles and endogenous ligands [18]. One of these receptors is the MR which recognizes extracellular host and foreign substances with exposed terminal mannose residues. Through this binding, the MR mediates the internalization of a wide range of pathogens and host-derived molecules; participates in the resolution of inflammation; and mediates clearance of apoptotic cells, endogenous hydrolases, and peroxidases [6,57,68]. In this study we have demonstrated that 43^MR^ macrophage hybridoma cells express the 175 kDa MR. This MR appears to function similarly to the receptor in primary macrophages and is regulated by agents known to affect the expression of the MR [39,69,70]. To our knowledge this is the first description of a continuous human macrophage cell line that expresses a functional MR.

Several continuous monocyte and macrophages lines currently exist that have been used to study macrophage biology including HL60, U937, THP-1, and MonoMac cells [71-74]. The cell surface marker profile of these cell types varies substantially and none express the exact complement of surface markers that are seen on primary macrophage. One disadvantage of using these cell types as surrogates for primary macrophages is that none express the MR, and this lack of an appropriate cell line has limited the study of MR biology in human systems. Much of the work that has been carried out has been performed in rat or murine macrophage cell lines that may or may not represent an accurate picture of the biology of the receptor in humans [45,49,75]. The parental 43 hybridoma cell line was described by Sperber et al. [54] and was created by fusion of the U937 macrophage continuous cell line with primary monocyte-derived macrophages. The resultant cells expressed donor-derived MHC I molecules and several other macrophage cell surface markers, but did not express detectable levels of the MR. In the current study, cells were grown to high confluence and cell sorting was used to select a subpopulation of cells expressing very low levels of MR. After successive sorts we ultimately obtained a population of cells in which >95% of the cells were MR-positive, and capable of being passaged with high MR maintenance. The isolation and characterization of these cells is significant for extending the characterization of the MR to human systems.

In the current study several experimental methods were employed to demonstrate that the MR in 43^MR^ cells is equivalent to the 175 kDa MR found in primary human and rodent macrophages. First, using PCR products were amplified from both the N- and C-terminal regions of the MR, indicating that these cells express functional domains important in ligand binding and receptor-ligand trafficking. Second, in immunoblot analysis using polyclonal anti-MR antibodies, a 175 kDa MR was observed as previously reported for the rat and human MR [49,76]. These findings are consistent with observations suggesting that concentrations of MR in rodent macrophages can be as much as 10 times greater than those found in human. Third, confocal imaging demonstrated a receptor expressed on the surface and in cytoplasmic compartments that is consistent with previous descriptions of MR localization [77]. In addition to immunoblot analysis illustrating a receptor of the same molecular weight and cellular distribution seen by confocal analysis, data from the surface binding and uptake assays showed ligand binding and internalization equivalent to results previously reported for primary human macrophages [31].

Another well-documented property of the MR is the correlation of expression with the functional state of the macrophage. Previous studies have shown that agents and cytokines such as dexamethasone, IL-4 and GM-CSF that promote a mature “deactivated” macrophage phenotype result in the induction of MR expression and altered MR. For example, Cowan et al. reported that treatment with dexamethasone increased MR expression on rodent macrophages by as much as 2.5 fold in a time and dose-dependent fashion [69]. Chakraborty et al. reported that dexamethasone-induced increases in the MR resulted in increased uptake of *Leishmania* parasites [29], and Zhu et al. demonstrated that dexamethasone treatment increased clearance of glucocerebrocidase by the MR [78]. In data not presented, we examined the effect of positive regulators (dexamethasone, GM-CSF, and IL-4) on MR expression in 43^MR^ cells. Dexamethasone at 0.1 μg/ml increased MR expression to approximately 130% of control, slightly under the previously reported increase seen in rat macrophages (150-200%: [42,69]). These results suggest that the MR in 43^MR^ cells can be positively regulated by dexamethasone as previously reported, and therefore provide further evidence that the MR expressed by 43^MR^ cells is functionally similar to the MR found in primary human macrophages.

The macrophage MR plays a key role in a number of innate host defense activities, including mediating entry of a variety of important human pathogens and clearance of harmful extracellular hydrolases. In the current study we demonstrated that two particle ligands previously shown to be internalized via the MR - *C. albicans* and the Gram positive bacteria *S. aureus –* were internalized by 43^MR^ cells, suggesting that the MR on these cells functions as a phagocytic receptor for these organisms.

Finally, we demonstrate the capacity of these cells to express exogenous proteins via transient transfection. In this study and in others we have successfully transiently expressed viral proteins into these cells using several different transfection preparations. The capacity for transient transfection allows for the introduction of proteins to study MR trafficking and its interaction with pathogen proteins.

## Conclusions

Understanding the molecular and cell biology of this receptor could be key to developing strategies for containing infection and controlling inflammation. However, lack of appropriate human cellular models that are available in large numbers and easily transfectable has hampered progress in this area. In the current study we have described a new human macrophage cell line developed by fusion of a human monocytic cell line (U937) and human monocyte-derived macrophages that express a functional MR on its surface, binds traditional MR ligands, and internalizes these ligands appropriately. In addition these cells can be transfected to a high efficiency, and are capable of expressing high levels of exogenous DNA. These cells therefore provide a powerful tool for the study of MR biology with respect to its role in innate immunity, and the role of the MR and the macrophage in the pathogenesis of disease.

## Competing interests

The authors declare that they have no competing interests.

## Authors’ contribution

DJV designed and carried out the studies on endocytosis, confocal microscopy, phagocytosis assays, and statistical analysis. SV participated in the flow cytometry experiments. VLS participated in the design and coordination of the studies. All authors approved the final manuscript.
